# (2*E*)-3-(4-Chloro­phen­yl)-1-(4-hy­droxy­phen­yl)prop-2-en-1-one

**DOI:** 10.1107/S160053681100701X

**Published:** 2011-03-02

**Authors:** Jerry P. Jasinski, Ray J. Butcher, H. S. Yathirajan, B.K. Sarojini, V. Musthafa Khaleel

**Affiliations:** a Department of Chemistry, Keene State College, 229 Main Street, Keene, NH 03435-2001, USA; b Department of Chemistry, Howard University, 525 College Street NW, Washington, DC 20059, USA; c Department of Studies in Chemistry, University of Mysore, Manasagangotri, Mysore 570 006, India; d Department of Chemistry, P. A. College of Engineering, Mangalore, 574 153, India

## Abstract

In the title compound, C_15_H_11_ClO_2_, the dihedral angle between the mean planes of the chloro­benzene and hy­droxy­benzene rings is 6.5 (6)°. The mean plane of the prop-2-en-1-one group makes an angle of 18.0 (1)° with the hy­droxy­phenyl ring and 11.5 (1)° with the chloro­phenyl ring. The crystal packing is stabilized by inter­molecular O—H⋯O hydrogen bonds, weak C—H⋯O, C—H⋯π and π–π stacking inter­actions [centroid–centroid distances = 3.7771 (7) and 3.6917 (7) Å].

## Related literature

For the biological properties of chalcones, see: Nowakowska (2007 ▶) and for their role in tubulin polymerization inhibition, see: Edwards *et al.* (1989 ▶). For related structures, see: Jasinski *et al.* (2010 ▶, 2011*a* ▶,*b* ▶); Butcher *et al.* (2007*a* ▶,*b* ▶); Narayana *et al.* (2007 ▶); Sarojini *et al.* (2007*a* ▶,*b* ▶). For standard bond lengths, see: Allen *et al.*, (1987 ▶).
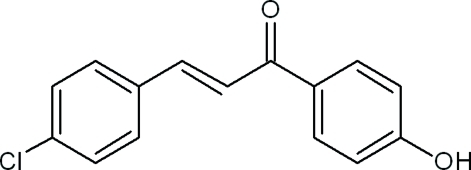


## Experimental

### 

#### Crystal data

C_15_H_11_ClO_2_
*M* *_r_* = 258.69
Monoclinic, 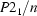

*a* = 7.3570 (2) Å
*b* = 15.6450 (5) Å
*c* = 10.4954 (3) Å
β = 90.518 (3)°
*V* = 1207.97 (6) Å^3^
*Z* = 4
Mo *K*α radiation
μ = 0.31 mm^−1^
*T* = 200 K
0.51 × 0.45 × 0.36 mm

#### Data collection

Oxford Diffraction Gemini diffractometer
Absorption correction: multi-scan (*CrysAlis RED*; Oxford Diffraction, 2007 ▶) *T*_min_ = 0.984, *T*_max_ = 1.000
10126 measured reflections
4020 independent reflections
2924 reflections with *I* > 2σ(*I*)
*R*_int_ = 0.022

#### Refinement

*R*[*F* ^2^ > 2σ(*F* ^2^)] = 0.041
*wR*(*F* ^2^) = 0.115
*S* = 1.07
4020 reflections
164 parameters
H-atom parameters constrained
Δρ_max_ = 0.38 e Å^−3^
Δρ_min_ = −0.19 e Å^−3^

### 

Data collection: *CrysAlis PRO* (Oxford Diffraction, 2007 ▶); cell refinement: *CrysAlis PRO*; data reduction: *CrysAlis RED* (Oxford Diffraction, 2007 ▶); program(s) used to solve structure: *SHELXS97* (Sheldrick, 2008 ▶); program(s) used to refine structure: *SHELXL97* (Sheldrick, 2008 ▶); molecular graphics: *SHELXTL* (Sheldrick, 2008 ▶); software used to prepare material for publication: *SHELXTL*.

## Supplementary Material

Crystal structure: contains datablocks global, I. DOI: 10.1107/S160053681100701X/sj5108sup1.cif

Structure factors: contains datablocks I. DOI: 10.1107/S160053681100701X/sj5108Isup2.hkl

Additional supplementary materials:  crystallographic information; 3D view; checkCIF report

## Figures and Tables

**Table 1:** Hydrogen-bond geometry (Å, °) *Cg*1 is the centroid of the C1–C6 ring.

*D*—H⋯*A*	*D*—H	H⋯*A*	*D*⋯*A*	*D*—H⋯*A*
O1—H1*O*⋯O2^i^	0.84	1.83	2.6556 (12)	167
C6—H6*A*⋯O1^ii^	0.95	2.57	3.5070 (13)	169
C11—H11*A*⋯O1^ii^	0.95	2.55	3.3382 (15)	141
C14—H14*A*⋯*Cg*1^iii^	0.95	2.79	3.7090 (14)	163

Symmetry codes: (i) 
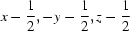
; (ii) 
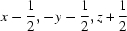
; (iii) 
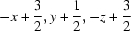
.

**Fig. 1.**
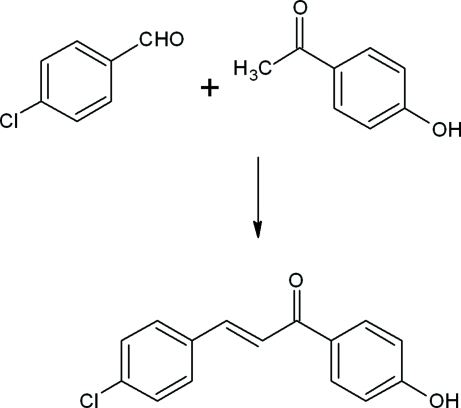
Reaction scheme for the preparation of (I).

**Fig. 2.**
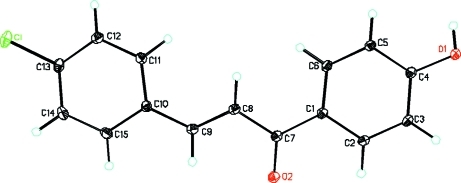
Molecular structure of the title compound showing the atom labeling scheme and 50% probability displacement ellipsoids.

**Fig. 3.**
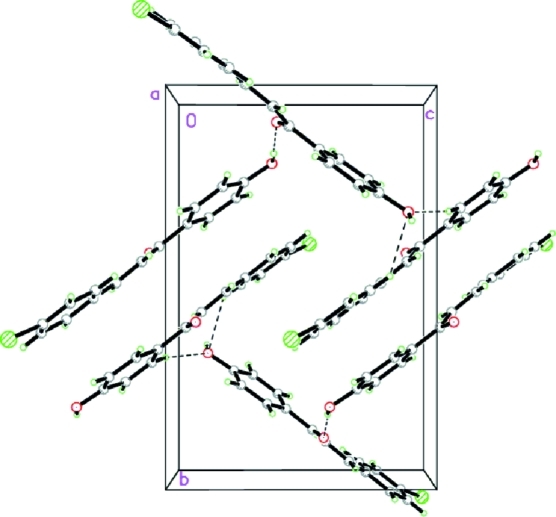
Packing diagram of the title compound viewed down the a axis. Dashed lines indicate O—H···O hydrogen bonds and weak C—H···O intermolecular interactions.

## supplementary crystallographic information

### Comment

Chalcones, or 1,3-diaryl-2-propen-1-ones, belong to the flavonoid family. Chemically they consist of open-chain flavonoids in which the two aromatic rings are joined by a three-carbon α,β-unsaturated carbonyl system. The radical quenching properties of the phenolic groups present in many chalcones have raised interest in using the compounds or chalcone rich plant extracts as drugs or food preservatives. Chalcones have been reported to possess many useful properties, including anti-inflammatory, antimicrobial, antifungal, antioxidant, cytotoxic, antitumor and anticancer activities (Nowakowska, 2007). Certain chalcone derivatives are reported to inhibit the polymerization of tubulin to form microtubules and are therefore antimitotic agents which can be used as antigout agents. The chalcone derivatives are also reported to inhibit the destruction of the myelin sheath in the central nervous system of multiple sclerosis patients and are thus useful in controlling the progressive nature of the disease (Edwards *et al.*, 1989).

In a continuation of our studies on the crystal structures of chalcones (Jasinski *et al.*, 2010, 2011*a*, 2011*b*), we report here the synthesis and crystal structure of the title compound, C_15_H_11_ClO_2_, (I), Fig. 2. The dihedral angle between the mean planes of the chlorobenzene and hydroxybenzene rings is 6.5 (6)°. The mean plane of the prop-2-en-1-one group, the active site in this molecule, makes angles of 18.0 (1)° with the hydroxy benzene and 11.5 (1)° with the chlorobenzene rings, respectively. Bond lengths are normal (Allen *et al.*, 1987) and correspond to those observed in related compounds (Butcher *et al.*, 2007*a*, 2007*b*; Narayana *et al.*, 2007; Sarojini *et al.*, 2007*a*, 2007*b*). Crystal packing is stabilized by O—H···O hydrogen bonds, weak C—H···O, C—H···*Cg*π—ring (Table 1) and π—π intermolecular stacking interactions (Table 2 & Fig. 3).

### Experimental

4-Hydroxyacetophenone (1.36 g, 0.01 mol) was mixed with 4-chlorobenzaldeyde (1.40 g, 0.01 mol) and dissolved in ethanol (20 ml) (Fig. 1). To this solution 4 ml of KOH (50%) (10 mL) was added at 0°C. The reaction mixture was stirred for 4 h and poured on to crushed ice. The pH of this mixture was adjusted to 3–4 with 2 *M* HCl aqueous solution. The resulting crude yellow solid was filtered, washed successively with dilute HCl solution and distilled water and finally recrystallized from ethanol (95%) to give the pure chalcone. Crystals suitable for *x*-ray diffraction studies were grown by the slow evaporation of the solution of the compound in acetone. M.P:419 K. Composition: Found (Calculated) for C_15_H_11_ClO_2_, C: 69.53 (69.64); H: 4.26 (4.29).

### Refinement

The hydroxyl hydrogem (H1O) was located by a Fourier map, fixed at 0.84 Å and refined using the riding model. All of the remaining H atoms were placed in their calculated positions and then refined using the riding model with Atom—H lengths of 0.95Å (CH). Isotropic displacement parameters for these atoms were set to 1.18–1.21 (CH) or 1.18 (OH) times *U*_eq_ of the parent atom.

### Crystal data

**Table:**

C_15_H_11_ClO_2_	*F*(000) = 536
*M**_r_* = 258.69	*D*_x_ = 1.422 Mg m^−^^3^
Monoclinic, *P*2_1_/*n*	Mo *K*α radiation, λ = 0.71073 Å
Hall symbol: -P 2yn	Cell parameters from 4738 reflections
*a* = 7.3570 (2) Å	θ = 4.7–32.4°
*b* = 15.6450 (5) Å	µ = 0.31 mm^−^^1^
*c* = 10.4954 (3) Å	*T* = 200 K
β = 90.518 (3)°	Irregular chunk, colorless
*V* = 1207.97 (6) Å^3^	0.51 × 0.45 × 0.36 mm
*Z* = 4	

### Data collection

**Table:**

Oxford Diffraction Gemini diffractometer	4020 independent reflections
Radiation source: fine-focus sealed tube	2924 reflections with *I* > 2σ(*I*)
graphite	*R*_int_ = 0.022
Detector resolution: 10.5081 pixels mm^-1^	θ_max_ = 32.6°, θ_min_ = 4.7°
φ and ω scans	*h* = −10→10
Absorption correction: multi-scan (*CrysAlis RED*; Oxford Diffraction, 2007)	*k* = −23→17
*T*_min_ = 0.984, *T*_max_ = 1.000	*l* = −11→15
10126 measured reflections	

### Refinement

**Table:**

Refinement on *F*^2^	Primary atom site location: structure-invariant direct methods
Least-squares matrix: full	Secondary atom site location: difference Fourier map
*R*[*F*^2^ > 2σ(*F*^2^)] = 0.041	Hydrogen site location: inferred from neighbouring sites
*wR*(*F*^2^) = 0.115	H-atom parameters constrained
*S* = 1.07	*w* = 1/[σ^2^(*F*_o_^2^) + (0.0648*P*)^2^] where *P* = (*F*_o_^2^ + 2*F*_c_^2^)/3
4020 reflections	(Δ/σ)_max_ < 0.001
164 parameters	Δρ_max_ = 0.38 e Å^−^^3^
0 restraints	Δρ_min_ = −0.18 e Å^−^^3^

### Special details

**Table:**

Geometry. All e.s.d.'s (except the e.s.d. in the dihedral angle between two l.s. planes) are estimated using the full covariance matrix. The cell e.s.d.'s are taken into account individually in the estimation of e.s.d.'s in distances, angles and torsion angles; correlations between e.s.d.'s in cell parameters are only used when they are defined by crystal symmetry. An approximate (isotropic) treatment of cell e.s.d.'s is used for estimating e.s.d.'s involving l.s. planes.
Refinement. Refinement of *F*^2^ against ALL reflections. The weighted *R*-factor *wR* and goodness of fit *S* are based on *F*^2^, conventional *R*-factors *R* are based on *F*, with *F* set to zero for negative *F*^2^. The threshold expression of *F*^2^ > σ(*F*^2^) is used only for calculating *R*-factors(gt) *etc*. and is not relevant to the choice of reflections for refinement. *R*-factors based on *F*^2^ are statistically about twice as large as those based on *F*, and *R*- factors based on ALL data will be even larger.

### Fractional atomic coordinates and isotropic or equivalent isotropic displacement parameters (Å^2^)

**Table:**

	*x*	*y*	*z*	*U*_iso_*/*U*_eq_	
Cl	0.13335 (5)	0.12309 (2)	0.03417 (3)	0.03910 (12)	
O1	0.83226 (11)	−0.31714 (6)	−0.88736 (8)	0.0269 (2)	
H1O	0.7288	−0.3393	−0.8952	0.032*	
O2	1.03166 (11)	−0.08981 (6)	−0.40899 (9)	0.0300 (2)	
C1	0.86149 (15)	−0.16357 (7)	−0.56445 (10)	0.0217 (2)	
C2	1.01626 (15)	−0.18219 (7)	−0.63990 (11)	0.0233 (2)	
H2A	1.1307	−0.1587	−0.6162	0.028*	
C3	1.00442 (15)	−0.23337 (7)	−0.74638 (11)	0.0240 (2)	
H3A	1.1090	−0.2440	−0.7964	0.029*	
C4	0.83821 (15)	−0.26923 (7)	−0.77995 (11)	0.0218 (2)	
C5	0.68456 (15)	−0.25387 (8)	−0.70399 (11)	0.0249 (2)	
H5A	0.5718	−0.2800	−0.7253	0.030*	
C6	0.69667 (15)	−0.20111 (8)	−0.59876 (11)	0.0250 (2)	
H6A	0.5916	−0.1903	−0.5494	0.030*	
C7	0.88030 (15)	−0.10548 (7)	−0.45464 (11)	0.0228 (2)	
C8	0.71613 (16)	−0.06563 (8)	−0.39876 (11)	0.0257 (2)	
H8A	0.5998	−0.0789	−0.4335	0.031*	
C9	0.72854 (16)	−0.01152 (7)	−0.30074 (11)	0.0244 (2)	
H9A	0.8482	0.0042	−0.2753	0.029*	
C10	0.57985 (15)	0.02682 (7)	−0.22700 (11)	0.0232 (2)	
C11	0.39739 (16)	0.00729 (8)	−0.25006 (12)	0.0269 (3)	
H11A	0.3655	−0.0270	−0.3215	0.032*	
C12	0.26083 (16)	0.03728 (8)	−0.16994 (12)	0.0286 (3)	
H12A	0.1376	0.0221	−0.1856	0.034*	
C13	0.30593 (17)	0.08902 (8)	−0.06791 (11)	0.0267 (3)	
C14	0.48422 (18)	0.11178 (8)	−0.04434 (12)	0.0296 (3)	
H14A	0.5144	0.1483	0.0250	0.036*	
C15	0.61975 (17)	0.08026 (8)	−0.12423 (12)	0.0277 (3)	
H15A	0.7427	0.0957	−0.1080	0.033*	

### Atomic displacement parameters (Å^2^)

**Table:**

	*U*^11^	*U*^22^	*U*^33^	*U*^12^	*U*^13^	*U*^23^
Cl	0.0429 (2)	0.0418 (2)	0.03272 (18)	0.01436 (15)	0.01185 (14)	0.00176 (13)
O1	0.0232 (4)	0.0314 (5)	0.0261 (4)	−0.0042 (3)	0.0043 (3)	−0.0055 (3)
O2	0.0223 (4)	0.0345 (5)	0.0330 (5)	0.0028 (4)	−0.0034 (3)	−0.0045 (4)
C1	0.0207 (5)	0.0229 (5)	0.0216 (5)	0.0009 (4)	0.0022 (4)	0.0022 (4)
C2	0.0175 (5)	0.0265 (6)	0.0259 (6)	−0.0006 (4)	0.0006 (4)	0.0022 (5)
C3	0.0190 (5)	0.0280 (6)	0.0250 (6)	0.0004 (4)	0.0053 (4)	0.0015 (4)
C4	0.0236 (5)	0.0218 (5)	0.0203 (5)	0.0008 (4)	0.0022 (4)	0.0034 (4)
C5	0.0185 (5)	0.0296 (6)	0.0265 (6)	−0.0049 (4)	0.0031 (4)	0.0009 (5)
C6	0.0204 (5)	0.0296 (6)	0.0250 (6)	−0.0003 (4)	0.0057 (4)	0.0003 (5)
C7	0.0219 (5)	0.0241 (6)	0.0226 (5)	0.0012 (4)	0.0013 (4)	0.0033 (4)
C8	0.0203 (5)	0.0312 (6)	0.0257 (6)	0.0018 (5)	−0.0001 (4)	−0.0020 (5)
C9	0.0221 (5)	0.0250 (6)	0.0262 (6)	−0.0004 (4)	−0.0004 (4)	0.0004 (5)
C10	0.0243 (5)	0.0210 (5)	0.0241 (5)	0.0009 (4)	0.0001 (4)	−0.0003 (4)
C11	0.0265 (6)	0.0274 (6)	0.0267 (6)	0.0013 (5)	−0.0025 (5)	−0.0048 (5)
C12	0.0226 (6)	0.0319 (6)	0.0312 (6)	0.0027 (5)	−0.0010 (5)	−0.0002 (5)
C13	0.0311 (6)	0.0239 (6)	0.0251 (6)	0.0074 (5)	0.0041 (5)	0.0028 (5)
C14	0.0381 (7)	0.0244 (6)	0.0263 (6)	0.0012 (5)	−0.0021 (5)	−0.0048 (5)
C15	0.0273 (6)	0.0268 (6)	0.0290 (6)	−0.0029 (5)	−0.0031 (5)	−0.0029 (5)

### Geometric parameters (Å, °)

**Table:**

Cl—C13	1.7517 (12)	C7—C8	1.4846 (15)
O1—C4	1.3541 (14)	C8—C9	1.3348 (16)
O1—H1O	0.8400	C8—H8A	0.9500
O2—C7	1.2328 (14)	C9—C10	1.4734 (15)
C1—C6	1.3917 (16)	C9—H9A	0.9500
C1—C2	1.4230 (14)	C10—C15	1.3939 (16)
C1—C7	1.4733 (16)	C10—C11	1.3957 (17)
C2—C3	1.3770 (16)	C11—C12	1.3971 (16)
C2—H2A	0.9500	C11—H11A	0.9500
C3—C4	1.3879 (16)	C12—C13	1.3804 (18)
C3—H3A	0.9500	C12—H12A	0.9500
C4—C5	1.4098 (15)	C13—C14	1.3793 (18)
C5—C6	1.3811 (17)	C14—C15	1.3984 (17)
C5—H5A	0.9500	C14—H14A	0.9500
C6—H6A	0.9500	C15—H15A	0.9500
			
C4—O1—H1O	109.5	C9—C8—H8A	119.3
C6—C1—C2	117.99 (10)	C7—C8—H8A	119.3
C6—C1—C7	122.56 (10)	C8—C9—C10	128.11 (11)
C2—C1—C7	119.45 (10)	C8—C9—H9A	115.9
C3—C2—C1	121.71 (10)	C10—C9—H9A	115.9
C3—C2—H2A	119.1	C15—C10—C11	117.45 (10)
C1—C2—H2A	119.1	C15—C10—C9	119.90 (11)
C2—C3—C4	119.35 (10)	C11—C10—C9	122.49 (11)
C2—C3—H3A	120.3	C10—C11—C12	121.18 (11)
C4—C3—H3A	120.3	C10—C11—H11A	119.4
O1—C4—C3	117.18 (10)	C12—C11—H11A	119.4
O1—C4—C5	123.03 (10)	C13—C12—C11	119.66 (11)
C3—C4—C5	119.78 (10)	C13—C12—H12A	120.2
C6—C5—C4	120.52 (11)	C11—C12—H12A	120.2
C6—C5—H5A	119.7	C14—C13—C12	120.79 (11)
C4—C5—H5A	119.7	C14—C13—Cl	120.34 (10)
C5—C6—C1	120.59 (10)	C12—C13—Cl	118.84 (10)
C5—C6—H6A	119.7	C13—C14—C15	118.92 (12)
C1—C6—H6A	119.7	C13—C14—H14A	120.5
O2—C7—C1	120.32 (10)	C15—C14—H14A	120.5
O2—C7—C8	119.88 (11)	C10—C15—C14	121.95 (11)
C1—C7—C8	119.80 (10)	C10—C15—H15A	119.0
C9—C8—C7	121.37 (11)	C14—C15—H15A	119.0
			
C6—C1—C2—C3	2.13 (17)	C1—C7—C8—C9	−178.78 (11)
C7—C1—C2—C3	−177.42 (11)	C7—C8—C9—C10	−173.67 (11)
C1—C2—C3—C4	−1.28 (17)	C8—C9—C10—C15	177.89 (12)
C2—C3—C4—O1	178.21 (10)	C8—C9—C10—C11	2.43 (19)
C2—C3—C4—C5	−0.86 (17)	C15—C10—C11—C12	−2.62 (18)
O1—C4—C5—C6	−176.85 (11)	C9—C10—C11—C12	172.94 (12)
C3—C4—C5—C6	2.17 (18)	C10—C11—C12—C13	1.71 (19)
C4—C5—C6—C1	−1.29 (18)	C11—C12—C13—C14	0.26 (19)
C2—C1—C6—C5	−0.81 (17)	C11—C12—C13—Cl	−178.00 (10)
C7—C1—C6—C5	178.72 (11)	C12—C13—C14—C15	−1.18 (19)
C6—C1—C7—O2	162.67 (11)	Cl—C13—C14—C15	177.05 (10)
C2—C1—C7—O2	−17.81 (17)	C11—C10—C15—C14	1.68 (18)
C6—C1—C7—C8	−17.34 (17)	C9—C10—C15—C14	−174.00 (11)
C2—C1—C7—C8	162.18 (11)	C13—C14—C15—C10	0.19 (19)
O2—C7—C8—C9	1.20 (18)		

### Hydrogen-bond geometry (Å, °)

**Table:**

*D*—H···*A*	*D*—H	H···*A*	*D*···*A*	*D*—H···*A*
O1—H1O···O2^i^	0.84	1.83	2.6556 (12)	167
C6—H6A···O1^ii^	0.95	2.57	3.5070 (13)	169
C11—H11A···O1^ii^	0.95	2.55	3.3382 (15)	141
C14—H14A···Cg1^iii^	0.95	2.79	3.7090 (14)	163

Symmetry codes: (i) *x*−1/2, −*y*−1/2, *z*−1/2; (ii) *x*−1/2, −*y*−1/2, *z*+1/2; (iii) −*x*+3/2, *y*+1/2, −*z*+3/2.

### Table 2 Selected geometric parmeters (Å): Cg···Cg π stacking interactions, Cg1, Cg2 are the centroids of rings C1—C6 and C10—C15 [Symmetry codes: (i) 1-x, 2-y, 1-z; (ii) 1-x, 2-y, 2-z]

**Table:**

*Cg*I···*Cg*J	Cg···Cg (Å)	CgI Perp (Å)	Cgj Perp (Å)	Slippage (Å)
Cg1···Cg2^i^	3.7771 (7)	3.3144 (5)	3.4958 (5)	
Cg2···Cg2^ii^	3.6917 (7)	-3.3684 (5)	-3.3683 (5)	1.51 (1)
